# Genetic Dissection of the Major Quantitative Trait Locus (qSE11), and Its Validation As the Major Influence on the Rate of Stigma Exsertion in Rice (*Oryza sativa* L.)

**DOI:** 10.3389/fpls.2017.01818

**Published:** 2017-11-02

**Authors:** Md Habibur Rahman, Yingxing Zhang, Keqin Zhang, Md Sazzadur Rahman, Hirendra N. Barman, Aamir Riaz, Yuyu Chen, Weixun Wu, Xiaodeng Zhan, Liyong Cao, Shihua Cheng

**Affiliations:** ^1^Crop Genetics and Breeding, China National Rice Research Institute, Hangzhou, China; ^2^Department of Agricultural Extension, Ministry of Agriculture, Dhaka, Bangladesh; ^3^Plant Physiology Division, Bangladesh Rice Research Institute, Gazipur, Bangladesh

**Keywords:** stigma exsertion rate, genetic dissection, major QTL (qSE11), near isogenic line, hybrid rice

## Abstract

The rate of stigma exsertion (SE) is an important trait in rice breeding because the efficiency of hybrid rice seed production can be improved by increasing the percentage of stigmas that exsert. In this study, we developed a near isogenic line (NIL) from two parents, XieqingzaoB (XQZB) and Zhonghoi9308 (ZH9308), which have high and low SE rates in that order. In our previous study, we employed 75 chromosome segment substitution lines (CSSLs) and analyzed quantitative trait loci (QTLs) for their influence on SE rate. The single gene QTL (qSE11), which is located on chromosome 11, was responsible for this trait. In this study, we focused on one of the CSSLs (C65), namely, the NIL (qSE11XB). It contains an introgression segment of XQZB in the genetic background of ZH9308, and exhibits a significantly higher SE rate than that of the parents. We demonstrated that qSE11 regulated both the single and the dual SE rates. They both contribute to the total SE rate. Genetic analysis revealed that qSE11 acted as a single Mendelian factor and that the allele from XQZB increased the SE rate. The validity of our conclusions was established when C65 was used to develop secondary F2 (BC5F2) and F2:3 (BC5F2:3) populations by backcrossing to ZH9308, with subsequent selfing. We entered 3600 plants from the F_2_ population and 3200 from the F_2:3_ populations into a genetic dissection program and dissected the major QTL qSE11 to a 350.7-kb region located on chromosome 11. This study will contribute to the future isolation of candidate genes of SE and will play a vital role in future hybrid rice seed production programs.

## Introduction

Rice (*Oryza sativa* L.) is a major cereal grains contributing approximately 60% of the daily dietary requirement, fulfilling about 20 and 14% of the calorie and protein requirement, respectively, of the world’s population (Mahalingam et al., 2013). By 2030, rice production would have to increase at least 40% to feed the extra population increased of the rice consuming countries (Kush, 2005). Hybrid rice has been developed and commercialized because of its yield benefits of 10–20% over high-yielding varieties (HYVs). Consequently the use of hybrid varieties along with better cultivation and management package would contribute to the increase of rice about 6 tons per hectare. These increases in rice production contributed to the China’s total production and accomplished self-sufficiency and continued to the food supply (Cheng et al., 2007). Exploitation of hybrid vigor in rice and breeding for high-yielding hybrid are considered one of the most important advances of rice genetics after HVY development in agriculture and is an important step against food shortages caused by the increasing global population (Duvick, 1999; Virmani, 2003). With an increase in the frequency of stigma exsertion (SE) in female lines of hybrid rice, the seed-setting rate in hybrid seed production and yield of hybrid seed both increased (Zetian and Yanrong, 2011). Several phenotypic traits contribute to the seed production efficiency of hybrid rice, and SE is especially emphasized as major component in increasing pollination and seed set (Kato and Nimai, 1987). A high SE rate is expected to trap more pollen, improve cross-pollination, and increase the efficiency of hybrid seed production in rice. The exserted stigmas remained viable for approximately 4 days and could continue to accept pollination (Long and Shu, 2000; Tian et al., 2004).

Molecular marker-assisted selection (MAS) is a powerful tool to increase breeding efficiency, but much work remains to be done before this technique can be extended from the major genes for QTL identification. Li et al. (2014) reported that the SE rate is a complex quantitative trait and is controlled by polygenes. Moreover, Li et al. (2014) carried out genetic mapping for SE rate in rice and confirmed the effects of identified quantitative trait loci (QTLs). Lou et al. (2014) also performed QTL mapping for SE in rice by using two *indica* cytoplasmic male sterile (CMS) maintainers such as Huhan1B and K17B of F_2_ population. They have reported for single stigma exsertion (SSE), dual stigma exsertion (DSE), and total stigma exsertion (TSE) rates by constructing linkage map of 92 SSR markers.

Recently, with the progress of genomics and DNA marker technology, a number of QTL mapping studies have been carried out elsewhere for SE in rice by using different populations such as recombinant inbred lines (RILs) and advance backcross (AB) lines. Yamamoto et al. (2003) used a RIL of *japonica* × *indica* population, another studies by Uga et al. (2003) identified two QTLs for SE between a cross of Pei-Kuh (an *indica* origin), and W1944 (*Oryza rufipogon* Griff.) (a wild cultivar). To overcome the process of cumbersome QTL procedure and to get benefits at the same time Tanksley and Nelson (1996) proposed a different strategy by using advanced backcross QTL (AB-QTL), where important major effect QTLs are introgressed during QTL detection through backcrossing in to elite cultivar. This method initially used in tomato (Tanksley et al., 1996) and then to rice (Xiao et al., 1998; Moncada et al., 2001; Thomson et al., 2003, 2010). In addition to QTL mapping, association mapping also carried out to identify SE for rice. Yan et al. (2009) reported RM5, a simple sequence repeat (SSR) marker highly correlated with both the dual and SSE rates in rice while analyzing a minicore of 90 accessions with 109 SSR marker. Yan et al. (2009) also identified 15 QTLs for SE on chromosomes 1, 5, 6, 7, 8, 9, 10, and 11 when analyzing Guangluai-4 and the wild cultivar W1943 (*O*. *rufipogon*) segregating populations. Chromosome segment substituted lines (CSSLs) and near isogenic lines (NILs) derived from same recurrent genetic background except the substituted or introgressed segments can have the potentiality to partition the QTL into single Mendelian factors.

The current study report about the major QTL *qSE11* for SE rates, using the C65 introgression line and the NIL (*qSE11^XB^*) derived from parents XieqindzaoB (XQZB) and ZH9308. The allele derived from XQZB of the *qSE11* associated with a higher SE rate than that in the recipient parent. We dissected the major QTL (*qSE11*) and narrowed it to a 350.7-kb region on the rice chromosome 11.

## Materials and Methods

### Plant Materials

**FIGURE 1:**
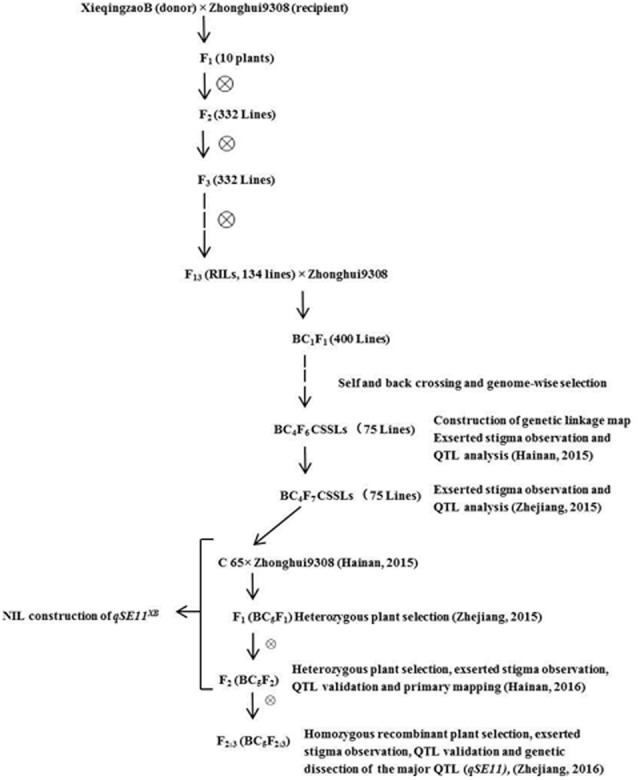
The detailed scheme for plant population development.

C65 is an introgression line, generated from XQZB as the donor parent and ZH9308 as the recurrent parent. C65 was backcrossed with ZH9308, and produced F_2_ (BC_5_F_2_) and F_2:3_ (BC_5_F_2:3_) populations by subsequent selfing. This allowed us to validate the QTLs for the SE rate. In this study, we developed the NIL (*qSE11^XB^*), which contained an introgression segment from XQZB in the genetic background of ZH9308. It had a significantly higher SE rate than its parents. The detailed scheme of the population development is shown in **Figure 1**.

### Field Experiments

C65 was crossed with ZH9308 at Hainan Island, China, in winter 2015; the F_1_ population and their parents were planted at Zhejiang, China, in summer 2015; the F_2_ population, the NIL (*qSE_*11*_^XB^*) and their parents were planted at Hainan Island in winter 2016. The F_2:3_ populations, the NIL (*qSE_*11*_^XB^*) and their parents were planted at Zhejiang in summer 2016. NILs were planted eight per rows and in a total of six rows per line. Spacing were maintained 30 cm between rows and 20 cm between plants. Standard crop management practices were followed.

### Determination of the Stigma Exsertion Rate

**FIGURE 2:**
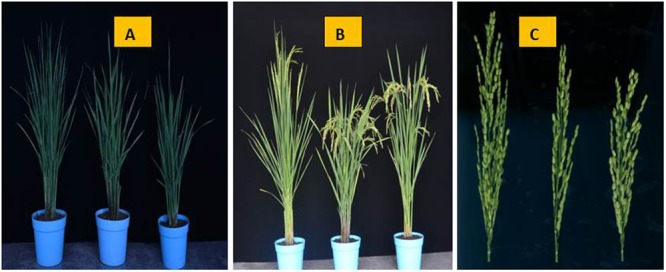
The phenotypes of the parents ZH9308, XQZB, and NIL (*qSE_*11*_^XB^*). **(A)** At 48 days after transplanting, the parental lines shown from the left to the right are ZH9308, XQZB, and NIL (*qSE_*11*_^XB^*); **(B)** at 82 days after transplanting, the parental lines shown from the left to the right are ZH9308, XQZB, and NIL(*qSE_*11*_^XB^*); **(C)** at 70 days after the transplanting, the panicles of the parental lines shown from the left to the right are ZH9308, XQZB, and NIL(*qSE_*11*_^XB^*).

**FIGURE 3:**
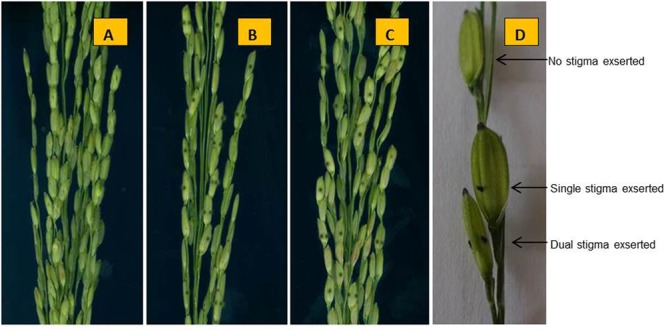
The phenotype of the stigma exsertion of the parents. **(A)** ZH9308 (stigmas are brown and not visible), **(B)** XQZB, **(C)** NIL (*qSE_*11*_^XB^*), and **(D)** examples of single, dual, and no stigma exsertion in a spikelet.

When stigmas remained outside even after closing of the lemma and palea then it is defined as exserted stigmas. The rate of SE classified into three traits: SSE, DSE, and TSE. At 5–7 days after heading, five panicles of each parent and NIL (*qSE_*11*_^XB^*) and three normal panicles from each plant in the F_2_ population in Hainan and the F_2:3_ populations in Zhejiang. The SE rates for both the conditions were observed when bottom spikelets of each panicle flowered. The calculations for SE percentages were followed according to the method Miyata et al. (2007) with modifications. The phenotypes of the parents ZH9308, XQZB, and NIL at 48 days after transplanting (**Figure 2A**), at 82 days after transplanting (**Figure 2B**), at 70 days after the transplanting, the panicles of the parental lines (**Figure 2C**). In short, the percent of SE is expressed as the rate of exserted stigmas based on the total stigmas of a panicle. The phenotype of the stigmas exsertion of the parents ZH9308 (**Figure 3A**), XQZB (**Figure 3B**), NIL (**Figure 3C**), and examples of single, dual, and no SE in a spikelet (**Figure 3D**); the counts of SE were converted by using the following formulas:

$$\begin{array}{c} \mathrm{SSE}\left(\%\right)=\left[\mathrm{SSE}/\left(\mathrm{SSE}+\mathrm{DSE}+\mathrm{No}\mathrm{stigma}\mathrm{exsertion}\right)\right]\times 100.\\ \mathrm{DSE}\left(\%\right)=\left[\mathrm{DSE}/\left(SSE+DSE+No\mathrm{stigma}\mathrm{exsertion}\right)\right]\times 100.\\ \mathrm{TSE}\left(\%\right)=\mathrm{SSE}\left(\%\right)+\mathrm{DSE}\left(\%\right). \end{array}$$

### NIL Development (*qSE*11*^XB^*)

In the previous study, the QTL (*qSE11)* was primarily mapped between RM167 and RM5704 on chromosome 11 by using a recombinant inbred population (RIL) derived from a cross between XQZB and Zhonhui9308 (Rahman et al., 2016). The BC_4_F_6_ population of the C65 line with the homozygous XQZB alleles, showing uniform SE, and the C65 line were selected for backcrossing with the recurrent parent ZH9308 to produce the F_2_ (BC_5_F_2_) and F_2:3_ (BC_5_F_2:3_) populations. The targeted *qSE11* region of C65 was homozygous for the XQZB alleles on chromosome 11. C65 was selected because it had the least amount of donor introgression with the *qSE11* region on chromosome 11. The NIL (*qSE11^XB^*) was developed on the basis of phenotypic performance, QTL validation, and genome-wide selection. Based on the genotypes of the XQZB alleles, one F_2_ (BC_5_F_2_) plant with homozygous XQZB regions surrounding the *qSE11* allele with a single segment was chosen as the NIL (*qSE11^XB^*). To screen the genetic background, 120 SSR markers that were evenly distributed on 12 chromosomes were selected from all polymorphic markers between XQZB and ZH9308 (McCouch et al., 2008).

### DNA Preparation, PCR Protocol, and Development of Molecular Markers

DNA was extracted from the fresh leaves of plants from the 3600 individuals of F_2_, 3200 individuals of F_2:3_ population, and their parents using the CTAB methods (Luo et al., 2005). During PCR every 12-μl reaction amount was reacted containing 1.6 μl of 21.0 ng/μl template extracted DNA, 0.26 μl of 1.1 pmol/μl dNTPs, 1.1 μl of 10× PCR buffer, 1.5 μl of 2.1 pmol/μl primer pairs, 0.07 μl of 5.1 U/μl Taq DNA polymerase, and 5.70 μl of ddH_2_O. During protocol consisted of primary denaturation step (95°C for 5.5 min), followed by 33 cycles of 95°C for 31 s, 54°C for 31 s, and 72°C for 1.1 min. The final reactions were completed with an extension step of 72.5°C for 8.5 min. The products of PCR were separated on the basis of electrophoresis 8.5% non-denaturing gels of polyacrylamide when silver staining visualized (Creste et al., 2001). The primer sequences of the 13 new molecular markers that were designed and contributed to this investigation are in **Table 1**.

**Table 1:** Marker name and sequences of InDel, SSR, and designed markers used in this study.

Markers	Forward primer (5′–3′)	Reverse primer (5′–3′)	Purpose
InD144	TGATGAGCTCTCACTTGTTGAAA	CGTACATTGGCTTATGTGATCTG	Linkage analysis
RM5704	AACGAATGATTAAACATCTA	AAGCAGAGTCAACATATTTA	Linkage analysis
SE 1-3	ACTGTCGCGGCTAAGATCCCTT	TGGGTTCGTCATGCTCCACAA	Genetic dissection
SE 3-1	TTTTAACCTGTTTCCCTTGCACAA	TGCCATTCAGTCCAATTTAGGATT	Genetic dissection
SE 3-3	GACCAATGGAAACGTCACCAGG	TTAGTATCTCGATGGCCACGATGG	Genetic dissection
SE 3-5	TAACCTGGAGGCATGTACACTT	CAGGCTGGAACTCCTCTGCAAACC	Genetic dissection
SE 3-7	CCTTCTGCAGCCTGGTCTTTCGG	GCCAAAACCGCTGTCCAAACCAA	Genetic dissection
SE 4-4	CTTCCCTCGAATTCTCGCTCCTT	GCACAACCTTCTGCGACAATTCCC	Genetic dissection
SE 5-5	GGCCGCCATGCATCTAGGTT	GAGGCAGGAGAAGCTTTACACACC	Genetic dissection
SE 6-5	CTCCTCCTTGGCTCTAGTCCTT	TAACCCTCATCTCTCTAGCCAGTT	Genetic dissection
SE 6-10	TGTAGCTAAAGTGAGCGTTGG	ACGCATTTGATCCCTAACAGAA	Genetic dissection
SE 7-2	TGGGCAGGTCATTTTAAGACACC	TGAGGCCATATATCGCACACAA	Genetic dissection
SE 8-1	TTGTAGGGTCATAACCGGATTT	TGGGCAAAGGCTATTATGCTCC	Genetic dissection
SE 8-2	TTTACCGACGACACCAGCTTTT	TCATCCGTGTTTCAGTAGGTTT	Genetic dissection
SE 10	GAGAAAGTTATTGCTAGCCTCAA	TTAGTGCCTTGTTATCAGCC	Genetic dissection

## Results

### Stigma Exsertion Performance XQZB, C65, ZH9308, and NIL (*qSE_*11*_^XB^*)

The phenotypic data and analyses of this study indicated that XQZB, C65, ZH9308, and NIL (*qSE11*^XB^) were significantly different in the single, dual, and TSE rates in the same row (**Table 2**), but the differences in the SSE and DSE between C65 and NIL (*qSE*11*^XB^*) were not significant. In the F_2_ segregating population, the mean SSE, DSE, and TSE were 19.92, 5.55, and 25.47. The highest values of the SSE, DSE, and TSE were 42.57, 14.57, and 54.88.

**Table 2:** The stigma exsertion rates of XQZB, C65, ZH9308, NIL (*qSE11^XB^*), and the F_2_ population developed from C65 and ZH9308.

Traits	XQZB ±*SE*	C65	ZH9308 ±*SE*	NIL (*qSE11*^XB^)	F_2_ population developed from C65 and ZH9308	
					Mean	Range
SSE	25.59 ± 1.00^a^	29.73 ± 0.54^b^	11.07 ± 0.42^c^	38.69 ± 0.49^bd^	19.92 ± 0.03	3.42–42.57
DSE	5.05 ± 0.25^a^	5.16 ± 0.12^b^	1.35 ± 0.11^c^	6.50 ± 0.19^bd^	5.55 ± 0.01	00–14.97
TSE	30.64 ± 0.92^a^	34.93 ± 0.59^b^	12.42 ± 0.41^c^	45.17 ± 0.40^d^	25.47 ± 0.04	3.85–54.88

*The superscript letters indicate statistically significant (*p* < 0.01) differences between the mean values within each row (Student’s *t*-test). SSE, single stigma exsertion rate; DSE, dual stigma exsertion rate; TSE, total stigma exsertion rate; NIL *(qSE11*^*XB*^), near isogenic line carrying the homozygous *qSE11* region from XQZB under the genetic background of ZH9308; SE, standard error.*

A highly significant QTL (*qSE11*), which had been predicted for the NIL (*qSE11^XB^*), was confirmed on the long arm of chromosome 11. The NIL phenotypic data (**Table 2**) showed that *qSE11* increased the percentage of exserted stigmas by 27, 5, and 32% in the SSE, DSE, and TSE, compared with ZH9308 on the basis of phenotypic evaluation. This result suggested that *qSE11* is the QTL responsible for high SE in the context of increasing the productivity of hybrid rice.

### Phenotypic Variation of Exserted Stigma in the F_2_ Segregating Population

**FIGURE 4:**
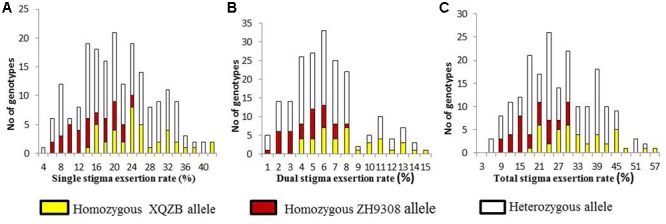
The frequency distribution of stigma exsertion rates in the F_2_ populations (**A** = single, **B** = dual, and **C** = total stigma exsertion rates). The yellow, red, and white colors indicate the genotypes of the homozygous XQZB allele, homozygous ZH9308 allele, and heterozygous allele, respectively.

The F_2_ individuals were classified SSE, DSE, and TSE based on the progeny test. The frequency of the SSE (**Figure 4A**) and TSE (**Figure 4C**) exhibited continuous variation and followed a normal distribution. The DSE (**Figure 4B**) showed a bimodal distribution, with a significant deviation from the normal distribution. These results suggested that SE rates followed Mendelian law, with little background noise. The *qSE11* expression was comparably stable and had a significant effect on the SE rates.

The correlation coefficients of SSE, DSE, and TSE are presented in **Table 3**. The traits were significantly correlated with each other. The highest phenotypic correlation was observed between the SSE and TSE (*r* = 0.989^∗∗^), followed by the DSE and TSE (*r* = 0.907^∗∗^) and the SSE and DSE (*r* = 0.833^∗∗^) in the F_2_ segregating population. This correlation indicated that the lines with a higher SSE rate were also more likely to exhibit increased DSE, which ultimately increased the TSE rate. The major QTL (*qSE11*) for SE rate, with an log of the odds (LOD) value varying from 2.14 to 5.6, was distributed on chromosome 11, the phenotypic variance explained by each QTL ranged from 7.28 to 18.8% and additive effect ranges 1.16–4.14 (**Table 4**).

**Table 3:** Correlation (Pearson) coefficients among the traits SSE, DSE, and TSE in the F_2_ populations.

	SSE	DSE	TSE
SSE	1		
DSE	0.833^∗∗^	1	
TSE	0.989^∗∗^	0.907^∗∗^	1

*^∗∗^, correlation is significant at the 0.01 level (two-tailed). SSE, single stigma exsertion rate; DSE, dual stigma exsertion rate; TSE, total stigma exsertion rate.*

**Table 4:** The putative major quantitative trait loci (QTLs) for the stigma exsertion rates detected in the CSSL and RIL (Rahman et al., 2016) populations derived from the parental lines XQZB and ZH9308.

Trait	QTL	Chr	Markers	Sources	CSSLs (75 Line)			RILs (134 Lines)		
					LOD	PVE (%)	A (%)	LOD	PVE (%)	A (%)
SSE	qSSE11	11	RM5704/RM286	XQZB	4.701	18.79	3.19	2.48	8.37	3.18
DSE	qDSE11	11	RM5704/RM286	XQZB	5.61	22.04	1.20	2.14	7.28	1.16
TSE	qTSE11	11	RM5704/RM286	XQZB	4.70	18.80	6.37	2.38	8.03	4.18

*A, the additive effect of each QTL; PVE, the phenotypic variance explained by each QTL; LOD, logarithm of odds; qSSE, QTL for the single stigma exsertion rate; qDSE, QTL for the dual stigma exsertion rate; qTSE, QTL for the total stigma exsertion rate.*

### Validation of QTL (*qSE11*) and Comparison of Stigma Exsertion

**FIGURE 5:**
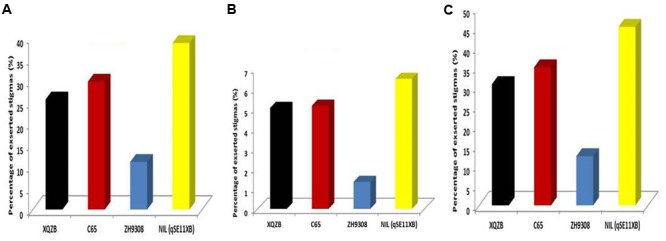
The comparison of stigma exsertion rates **(A)** SSE, **(B)** DSE, and **(C)** TSE among XQZB, C65, ZH9308, and NIL (*qSE_*11*_^XB^*) during the field evaluations for QTL validity.

In this study, QTL validation was performed in the appropriate region of the genome of the F_2_ and F_2:3_ progenies. The genotypes of F_2_ plants at the *qSE11* locus were easily measured based on the SE rates of their progeny. The SE rate of the recurrent parent ZH9308 was 11.07% (SSE), 1.35% (DSE), and (12.42), whereas the NIL phenotypic data were higher (38.69, 6.50, and 45.17%, respectively). Thus, the NIL had an increased rate of exsertion frequency of 27, 5, and 32% (SSE, DSE, and TSE), compared to the recurrent parent ZH9308. See **Figure 5** for a depiction of exsertion rates: single (**Figure 5A**), dual (**Figure 5B**), and total (**Figure 5C**). This result indicates that *qSE11* is responsible for the high SE rate in the NIL.

### The Genetic Background and the Major QTL (*qSE11*) Position

**FIGURE 6:**
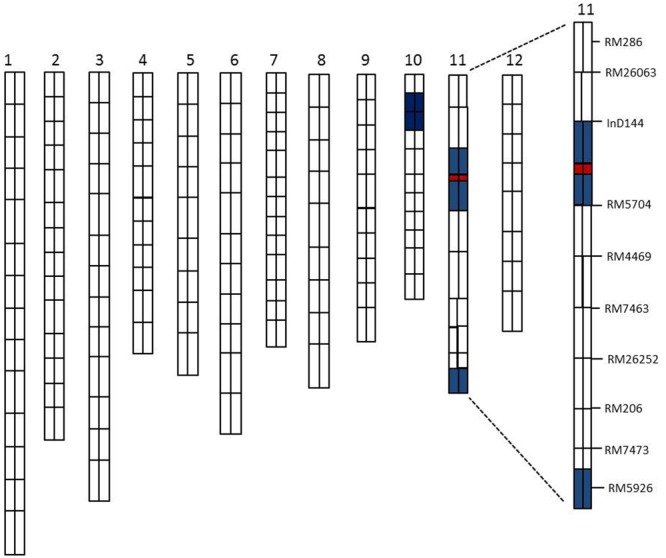
The rice chromosomal map; the white regions indicate the ZH9308 genetic background and the blue regions indicate the XQZB allele introgressions. The red regions indicate the major QTL (*qSE11*) locus.

A total of 134 RILs were used for primary QTLs mapping of the SE rate and 198 SSR markers for genetic linkage map (Rahman et al., 2016). The basis of the linkage map of the CSSL population was the previously established polymorphisms between the parents in this study (XQZB and ZH9308). The F_2_ population of C65 was the source for the major QTL (*qSE11*) confirmation and the F_2:3_ populations enabled the narrowing of the region. The genetic dissection map was prepared on the basis of genetic analysis and phenotypic performance of the SE rate. The white regions indicate the genetic background of ZH9308, blue regions indicate the introgressions of the XQZB allele, and the red regions indicate the major QTL (*qSE11*) locus in the chromosomal map of the CSSL population (**Figure 6**).

### Homozygous Recombinant Plant Selection

**FIGURE 7:**
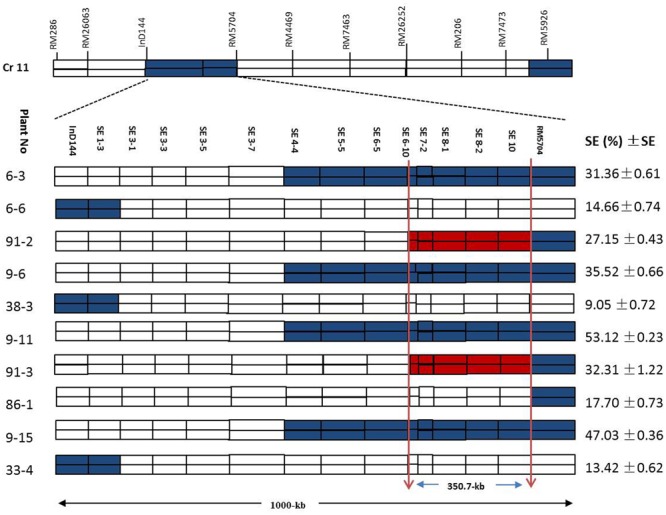
The genetic dissection of the homozygous recombinant plants in the F_2:3_ populations in the 1000-kb region between the markers InD144 and RM5704 on chromosome 11, showing the positions of 13 newly sequenced markers; the blue regions indicate XQZB allele introgressions. The red regions indicate the major QTL (*qSE11*) locus, and the white regions indicate the ZH9308 genetic background. SE (%), stigma exsertion rates; SE, standard error.

**Figure 7** shows a chromosomal dissection in which each homozygous recombinant plant contains a single or several donor (XQZB) segments (blue), recipient (ZH9308) segments (white), and a *qSE11* allele (red) on chromosome 11. The lengths of the substituted chromosome segments in the homozygous plants were estimated on the basis of the marker responses. Plants of the F_2_ generation (*n* = 3600) contributed to genetic dissection needed to screen the heterozygous plants. The homozygous plants were identified on the basis of the InDel144 and RM5704 marker response. The targeted region of *qSE11* was delimited to 1000 kb. To narrow the region, we sequenced 13 new molecular markers (**Table 1**). Genetic analysis was conducted with the new markers in 3200 individuals of the F_2:3_ populations. We evaluated the gene effect by employing the new markers in the targeted region, and selected the homozygous recombinant plants. We then validated the phenotypic performance of the SE rate (SE %), which varied from 9.05 to 53.12% (**Figure 7**) in the homozygous recombinant plants. On the basis of the genetic and phenotypic analysis, the targeted region was finally narrowed to an area between the SE6-10 and SE10 markers.

**FIGURE 8:**
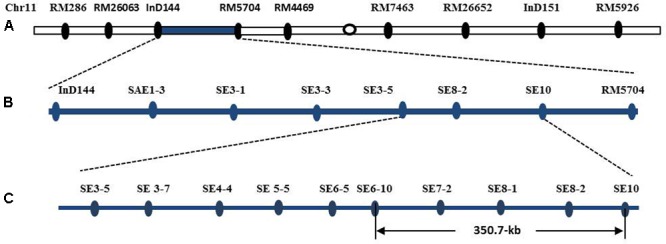
The chromosomal and physical maps of the qSE11. **(A)** The conventional QTL mapping in the CSSL population placed qSE11 on chromosome 11 between the markers InD144 and RM5704, with the adjacent markers RM286 and RM4469, respectively. **(B)** Primary mapping of the qSE11 based on the F_2_ and the derived F_2:3_ populations; the allele was mapped to the region between markers SE3-5 and SE8-2. **(C)** The physical location of the genetic dissection of qSE11 within a 350.7-kb region between the markers SE6-10 and SE10.

The introgression segment was located between the markers InD144 and RM5704 (**Figure 8A**), so that they could be employed in the primary mapping process. We designed and developed 73 pairs of sequence-tagged markers in the interval between InD144 and RM5704 to delimit the gene to a smaller region for the genetic dissection of the *qSE11* gene. Only 13 of these markers were identified as polymorphic between the parents (**Table 1**). Further recombinant screening with the newly designed markers showed that the gene was located between SE3-5 and SE10 (**Figure 8B**), and, with these newly designed markers and high-resolution genetic dissection analysis with 3200 individuals from F_2:3_ populations, the *qSE11* was finally narrowed to a 350.7 kb region between the SE6-10 and SE10 markers (**Figure 8C**).

## Discussion

Enhancing our knowledge of SE, a major focus of the hybrid rice breeding programs that are needed to produce for high yielding genotypes that will feed the ever increasing human population of our planet (Tang et al., 2013). A small number of SE QTL has previously been detected in rice chromosomes. Yamamoto et al. (2003) reported nine QTLs on the chromosomes 3, 4, 6, 8, 11, and 12. A large phenotypic effect was detected at *qES3*, located in the centromeric region (R1002) on chromosome 3. Yan et al. (2009) reported 15 QTLs on chromosomes 1, 5, 6, 7, 8, 9, 10, and 11. Two markers RM5704 and RM167 were identified on chromosome 11 that associated with *qSSE11, qDSE11*, and *qTSE11* those are similar to current study. Numerous studies have described QTLs associated with SSE, DSE, and TSE in rice. Specifically two QTLs for percentage of exserted stigmas were located on chromosomes 2 and 3 but the only QTL for dual stigma exsertation located on chromosome 2 (Li et al., 2003); three QTLs were found to influence percentage of single exerted stigma and three QTLs were also detected to influence percentage of dual exerted stigma on chromosomes 1, 2, 5, and 8 (Hu et al., 2009); seven QTLs for SSE rate were detected on chromosomes 1, 3, 6, 7, 9, and 12; four QTLs for DSE rate were detected on chromosomes 1, 6, and 10 (Li et al., 2014); and one QTLs for percentage of single exerted stigma, three QTLs for percentage of dual exerted stigma, and one QTLs for percentage of total exerted stigma were detected on chromosomes 5, 6, and 7 (Lou et al., 2014).

The increasing human population, reduction of cultivable land area, and environmental changes pose great challenges to rice production (Takeda and Matsuoka, 2008; Zhu et al., 2011). MAS is an effective technique in molecular breeding protocols that reduce the cost, improve the efficiency and accuracy of selecting the desired traits (Ribaut and Hoisington, 1998; Young, 1999). The exploitation of DNA markers focuses upon the identification of a desired trait that is required to characterize a specific phenotype. MAS can be applied after fulfilling some basic conditions, and with a better understanding of the genetic basis of all desirable traits that are linked to target genes. During the past decade many successful crop improvements have been achieved by adopting MAS protocols. However, a few findings on quantitative trait improvement, especially on SE traits have been reported in rice. The instability of QTL expression and unavailability of reliable markers are two critical barriers to the large-scale utilization of MAS in high-yield hybrid rice breeding (Zhu et al., 2011).

The exserted stigma is fragile and can be easily damaged by environmental conditions during the flowering period, e.g., wind, water stress, and physical damage (Yan et al., 2009). Observation and counting, when the lower spikelets had flowered, were made with great care to avoid this kind of damage to the stigmas. Therefore, the data generated in the present study with this technique will provide an accurate description of SE in the genotypes we studied. Even though observations were made at two sites, that experienced a range of weather conditions, we believe that our sampling method was sufficiently robust and that location and climate did not influence the outcomes of our experiments.

Three significant QTLs were detected in the previous study and were considered to have segregated in the F_2_ populations. This may be the reason for the continuous variation of this trait (**Figure 4**). One major QTL (*qSE11*) was dissected in this study. The major QTL (*qSE11*) is located in the long arm region of rice chromosome 11. This sector consists of introgression segments from XQZB and the genetic background of ZH9308. The major QTL (*qSE11*) for SE rate behaved as a single Mendelian factor. The most significant effect of the presence of the *qSE11* allele was an increase in the rice SE rate. The expression of *qSE11* also altered the SSE, DSE, and TSE. ZH9308 has a low SE rate. On the basis of genotyping and phenotypic validation, it is clear that *qSE11* activates the appropriate alleles that produce a higher SE rate.

In the segregating F_2_ population, the *qSE11* region was heterozygous due to the q*SSE11, qDSE11*, and *qTSE11* alleles. The F_2_ progeny showed a very wide variation in SE, which suggested that *qSE11* influenced the variation in SE in this population. Selection of the homozygous recombinant plants validated the performance of SSE, DSE, and TSE. Specifically, *qSE11* is a novel gene.

The present study developed 13 new InDel markers that were applied for the genetic dissection of *qSE11* in F_2:3_ populations. The frequency distribution of the SE rate in F_2_ populations was discontinuous and an analysis of the tested progeny showed that it followed the Mendelian ratio (1:2:1) for single locus segregation. The major QTL *qSE11* was primarily mapped to a 1000-kb region between the markers InD144 and RM5704. Finally, we delimited the *qSE11* allele to a 350.7-kb region by using the newly developed 13 sequence-tagged markers.

## Conclusion

Commercial hybrid rice seed production can be improved by increasing the SE rate in the CMS line. In this study, we identified QTL (*qSE11*) which is a or the gene that controls this trait. We predicted and then confirmed that it is on the long arm of chromosome 11. The NILs that included QTL (*qSE11*) in their genomes had phenotypic improvements in terms of the frequency stigma exertion. There were increases of 27% (SSE), 5% (DSE), and 32% (TSE) compared to that of the recipient parent ZH9308. These results suggest that *qSE11* is a promising QTL for the development of maternal lines with a rate of stigma for inclusion in hybrid rice seed production.

The most important achievement in our study was narrowing down the location of *qSE11* to a 350.7-kb region. This information will be helpful for the future identification, isolation, and cloning of candidate genes. We also developed a number of tightly linked markers for *qSE11* as by-products of our genetic dissection. These markers are useful tools for marker-assisted introgression of the SE trait in hybrid rice breeding programs.

## Author Contributions

SC, LC, and YZ conceived and designed the study. MHR conducted the experiments and received help from KZ and AR. YZ, WW, and XZ guided the observation of the stigma exsertion rates. MSR, HNB, and YC analyzed the data and formatted the figures. MHR and YZ wrote the paper. LC and SC reviewed and edited the manuscript. Finally the manuscript was read by all the authors and approved.

## Conflict of Interest Statement

The authors declare that the research was conducted in the absence of any commercial or financial relationships that could be construed as a potential conflict of interest.
